# Potentially inappropriate medications according to STOPP-J criteria and risks of hospitalization and mortality in elderly patients receiving home-based medical services

**DOI:** 10.1371/journal.pone.0211947

**Published:** 2019-02-08

**Authors:** Chi-Hsien Huang, Hiroyuki Umegaki, Yuuki Watanabe, Hiroko Kamitani, Atushi Asai, Shigeru Kanda, Hideki Nomura, Masafumi Kuzuya

**Affiliations:** 1 Nagoya University Graduate School of Medicine, Department of Community Healthcare & Geriatrics, Showa-ku, Nagoya, Aichi, Japan; 2 E-Da Hospital, Department of Family Medicine, Jiaosu Village, Yanchao District, Kaohsiung City, Taiwan, R.O.C; 3 I-Shou University, School of Medicine for International Students, Jiaosu Village, Yanchao District, Kaohsiung City, Taiwan, R.O.C; 4 Nagoya University Hospital, Department of Hospital Pharmacy, Showa-ku, Nagoya, Aichi, Japan; 5 Sanei Clinic, Komaki, Aichi, Japan; 6 Minami Health-Medical Cooperative Kaname Hospital, Minami, Nagoya, Aichi, Japan; 7 Aichi Clinic, Tenpaku, Nagoya, Aichi, Japan; University of Malaya, MALAYSIA

## Abstract

**Background:**

Although potentially inappropriate medications (PIMs) have been linked to poor health outcomes, country-specific PIM criteria have not been compared. Thus, we compared the identification of PIMs between the Screening Tool for Older Person’s Appropriate Prescriptions for Japanese (STOPP-J) and the 2015 American Geriatrics Society Beers Criteria in elderly patients receiving home-based medical services.

**Methods:**

A 5-year prospective cohort study was conducted with 196 patients receiving home-based medical services. Data were collected using questionnaires and chart reviews and included detailed information on prescription medication. STOPP-J and the Beers Criteria were used to categorize PIM and non-PIM recipients. All-cause mortality and first hospitalization were compared using a multivariate Cox regression model.

**Results:**

PIMs were detected in 132 patients (67.3%) by STOPP-J and in 141 patients (71.9%) by the Beers Criteria, and the mean numbers of PIMs were 1.3 ± 1.3 and 1.2 ± 1.1, respectively. The three most frequently prescribed STOPP-J PIMs were hypnotics (26.8%), diuretics (25.6%), and NSAIDs (12.6%), compared with proton pump inhibitors (PPIs) (29.8%), hypnotics (26%), and NSAIDs (8.1%) according to the Beers Criteria. STOPP-J PIMs were associated with all-cause mortality (HR 3.01, 95% CI 1.37–6.64) and hospitalization (HR 1.91, 95% CI 1.17–3.09); neither was associated with Beers Criteria PIMs. Using a modified Beers Criteria (excluding PPIs), PIMs were correlated with first hospitalization (HR 1.91, 95% CI 1.17–3.09).

**Conclusions:**

PIMs categorized by STOPP-J are associated with hospitalization and mortality in Japanese patients receiving home-based medical services. PPIs, commonly used for acid-related diseases, do not seem to have deleterious effects on health outcomes. Country-oriented, medication-specific criteria would be of considerable clinical utility.

## Introduction

Multiple comorbidities with medication burden are common in the elderly population [1]. In addition, the concurrent use of potentially inappropriate medications (PIMs) has been associated with adverse drug reactions, disability, mortality, hospitalization, institutionalization to aged care facilities, and high health costs [2–4]. PIM use is not uncommon. A nationwide study conducted in Denmark showed that 29% of non-demented and 38.1% of demented community-dwelling individuals used PIMs according to Danish criteria [5]. Another large retrospective cohort study performed on an isolated island in Korea revealed that 88% of the community-dwelling elderly filled prescriptions for PIMs according to 2015 American Geriatrics Society (AGS) Beers Criteria [6]. For old-aged hospitalized patients, a systematic review determined that the prevalence of PIMs for dementia and non-dementia patients ranged from 53.2% to 89.8% with the Beers Criteria and from 30.4% to 97.1% with the Screening Tool of Older Person’s Prescriptions (STOPP) criteria [7]. A systematic review found that 16–54% of nursing home residents used PIMs according to the Beers Criteria, Holmes, or Healthcare Effectiveness Data and Information Set (HEDIS) criteria, with 9–27% of all participants using antipsychotics and benzodiazepine [1, 8–10]. Although the prevalence of PIM use varies widely in acute and chronic care settings, PIMs are worthy of further investigation and management.

Several hospital-based studies have revealed the harmful effects of PIMs categorized according to the Beers Criteria, including functional impairment and increased length of hospital stay [11, 12]. The above-mentioned population-based retrospective study in Korea demonstrated that older community-dwelling patients taking at least one PIM (Beers Criteria) were at greater risk of hospitalization (OR 2.25, 95% CI 2.09–2.44) [6]. Screening and early detection of PIMs are therefore mandatory for the provision of quality-based medical care. Several externally validated society guidelines and national evidence-based screening tools are now available, including the Beers Criteria from the US, and STOPP and EU(7)-PIM from Europe [13–15]. However, drug availability and classification systems are not uniform across countries [16], and there is limited overlap between drug indication and contraindication criteria for specific diseases. A systematic review revealed that only 4 categories of PIMs (44 drugs in total) were listed in common in 25 out of 36 available PIM criteria [16]. Accordingly, country-specific PIM criteria are required for daily clinical practice and continuity of care. As a result, the Japan Geriatrics Society developed “Guidelines for Medical Treatment and its Safety in the Elderly” in 2005 and updated them in 2016 to the latest version, the so-called Screening Tool for Older Person’s Appropriate Prescriptions for Japanese (STOPP-J) [17].

STOPP-J is a clinical practice guideline and consensus statement for the prescription of drugs to the Japanese elderly [17]. A working group conducted a systematic review based on clinical questions and keywords and decided the level of recommendation following the GRADE approach (Grading of Recommendations Assessment, Development, and Evaluation) [18]. After external reviews by other medical specialty societies, professional organizations, and comments from the public, the final version of the guidelines comprised two lists: List of drugs to be prescribed with special caution and List of drugs to consider starting. The former list, which corresponds to the 2015 Beers Criteria from the USA and to the STOPP criteria from Europe, was used to identify PIMs in the present study.

PIMs have been found to correlate with health outcomes including death and hospitalization in community-dwelling older persons [19, 20], nursing home residents [21, 22], dementia patients [23], hospitalized patients [24–26], and patients requiring palliative care [27]. Nevertheless, the harmful effects of PIMs have seldom been investigated in community-dwelling disabled patients receiving home care. Moreover, the association with health-related outcomes has not yet been investigated using the newly developed PIM criteria, STOPP-J.

The aim of this study was to explore the association between PIM use, as determined by STOPP-J, and health-related outcomes in patients receiving home-based medical services in Japan. To better understand the need for country-specific PIM criteria, we also included leading international PIM criteria for comparison. However, because most medicines mentioned in the STOPP criteria are not available in Japan [13], the 2015 Beers Criteria and STOPP-J were used to identify the impact of PIMs on hospitalization and mortality rates in the present study.

## Methods

### Study population

We established a prospective cohort study from December 2012 to December 2017 known as the Observational study of Nagoya Elderly with HOme Medical Care (ONE HOME study) [28]. Under the Japanese national health insurance and long-term care insurance systems, home-based medical services are available for disabled patients who have difficulty reaching a clinic or hospital [29]. Insurance covers regular visits (daily up to once every 2 weeks depending on medical needs) from physicians, nurses, care managers, social workers, occupational therapists, physical therapists, and dieticians. Care managers hold a care provider meeting to discuss care plans among multidisciplinary team members every 4 to 6 months. In this study, we recruited consenting patients older than 45 years who were receiving such home-based medical services from seven satellite hospitals and clinics of Nagoya University Hospital in Aichi Prefecture. A total of 196 patients were enrolled and the study was approved by the Institutional Review Board of Nagoya University Graduate School of Medicine. Written informed consent was obtained from all participants in the study.

### Data collection

Visiting nurses who were trained and certified in the initial stage of research conducted comprehensive face-to-face interviews and in-home assessments with the patients. After registration was completed, a trained nurse also reviewed hospital and clinic charts twice a year during the follow-up period to collect data on medical history, including medication lists, hospitalization and institutionalization, and mortality. Visiting physicians and nurses maintained all charts. To ensure the accuracy of drug information, visiting pharmacists and nurses checked the patients’ home drug diaries.

Baseline profiles including age and sex were obtained. Medical history was gauged using the Charlson Comorbidity Index (CCI), which considers disease number and severity [30]. Daily functional status was assessed with the Independence Scale of the Disabled Elderly (ISDE) [31] and the Barthel Index. In terms of the ISDE, all participants were categorized into three groups according to the clinical judgment of visiting physicians: independent (Rank J), pre-bedridden (Rank A), and bedridden (Rank B & C) [31]. The Barthel Index reflects ability in basic activities of daily living (0 points indicating complete dependence to 100 points indicating complete independence). In addition, the Mini Nutritional Assessment-Short Form (MNA-SF), which is used to assess food intake, weight loss, mobility, psychological stress or acute disease, neuropsychological problems, and body mass index, was administered to investigate nutritional status [32]. Patients were stratified by total MNA-SF score as having malnutrition (≤ 7 points), being at risk of malnutrition (8–11 points), or having a normal nutritional status (12–14 points) [33].

The trained nurse reviewed hospital and clinic medical records and compared them against the home drug diary to trace current drug lists at the registration date. The following data were recorded: category, name, number, dose, route, and administration time of all medications taken, including over-the-counter drugs and drugs prescribed by other medical facilities. Only long-term (> 2 weeks) medicines were candidates and topical medicines and “when required” (*pro re nata*) medicines were excluded. Patients were deemed as having polypharmacy if they took ≥ 5 different medications [34].

### PIM criteria

PIMs were assessed using the 2015 AGS Beers Criteria (general recommendation, independent of diagnosis) [15] and STOPP-J (considering the clinical indication) [17]. The same trained nurse categorized PIMs into 23 and 19 groups, respectively. Because many medicines listed in the Beers Criteria are not available in Japan, we mainly set the classification categories using STOPP-J. The STOPP-J categories are antipsychotics (first and second generation), hypnotics (barbiturates, benzodiazepines, non-benzodiazepine receptor agonists), antidepressants, sulpiride, antiparkinson drugs, steroids, antithrombotic drugs (antiplatelet drugs, anticoagulants), digitalis, diuretics, β-blockers, α-blockers, first-generation H1 receptor antagonists, H2 receptor antagonists, antiemetic drugs, laxatives, oral antidiabetic drugs, insulin, overactive bladder medications, and NSAIDs. Besides the aforementioned categories, the Beers Criteria include four additional classes: proton pump inhibitors (PPIs), anticonvulsants, dihydropyridine calcium channel blockers, and non-dihydropyridine calcium channel blockers. The active ingredients, brand names, and generic names of medicines were independently scrutinized by another pharmacist to ensure classification accuracy. Disagreements between the nurse and the pharmacist were resolved by a geriatrician.

### Follow-up

All participants were investigated throughout the study period aside from those who were institutionalized for more than 6 months or who died within the first year after recruitment. For participants who were admitted to hospital or facilities for less than 6 months, the follow-up visits were restarted after discharge.

### Statistical analysis

Baseline profile data including age, sex, household status, marital status, CCI, number of medications, functional status, and nutritional status are described as counts and percentages. The periods after enrollment to first hospitalization and death during follow-up were estimated for patients with or without PIMs categorized according to the Beers Criteria and STOPP-J. A multivariate Cox regression model was used to test for the association between PIMs, mortality, and hospitalization after adjustment for age, sex, CCI, Barthel Index, MNA-SF, and polypharmacy. All statistical analyses were conducted using SPSS for Windows software version 22.0 (IBM Corp., Armonk, NY, USA). A two-tailed *p* value < 0.05 was considered statistically significant.

## Results

### Population characteristics

A total of 196 patients were enrolled, with a mean age of 80.2 ± 10.4 years. The baseline profiles including household and marital status are shown in Table 1. Most patients were male (56.9%), married (56.8%), and lived with their spouse (88.6%). Patients, all of whom were judged to need home-based medical services because of disability, had multiple comorbidities (CCI score: 3.0 ± 2.2), low functional status (Barthel Index score: 48.4 ± 34.1), and malnutrition (MNA-SF score: 7.7 ± 3.0). The number of medications prescribed was 5.7 ± 3.3, and 121 patients (63.4%) regularly took more than five medicines daily. Hospitalization and death were observed in 100 (50.8%) and 47 (23.9%) patients, respectively. The period from enrollment to first hospitalization was 481.5 ± 450.9 days; the period from enrollment to death was 611.3 ± 499.6 days. At the end of the follow-up period, 47 patients (24%) had died and 100 patients (51%) had been admitted to hospital at least once.

**Table 1:** Baseline characteristics of disabled participants receiving home-based medical services in Japan.

Variable	Total (N = 196)
Age, years (mean ± SD)	80.2 ± 10.4
Age group, n (%)	
45–65 years	18 (9.2%)
66–85 years	112 (57.1%)
≥ 86 years	66 (33.7%)
Sex, n (%)	
Male	112 (56.9%)
Female	84 (43.1%)
Household status, n (%)	
Alone	22 (11.4%)
Not alone	174 (88.6%)
Marital status, n (%)	
Married	111 (56.8%)
Widow/widower	65 (33.5%)
Divorced	7 (3.8%)
Single	11 (5.9%)
CCI, scores (mean ± SD)	3.0 ± 2.2
Medications, n (mean ± SD)	5.69 ± 3.34
Polypharmacy (≥5 medications), n (%)	
No	70 (36.6%)
Yes	121 (63.4%)
Barthel index, score (mean ± SD)	48.4 ± 34.1
MNA-SF, scores (mean ± SD)	7.7 ± 3.0
Malnourished, n (%)	66 (33.5%)
At risk of malnutrition, n (%)	75 (38.1%)
Normal nutritional status, n (%)	17 (8.6%)
Serum albumin level, g/dL (mean ± SD)	3.5 ± 0.6

### PIMs according to STOPP-J and the Beers Criteria

**Fig 1:**
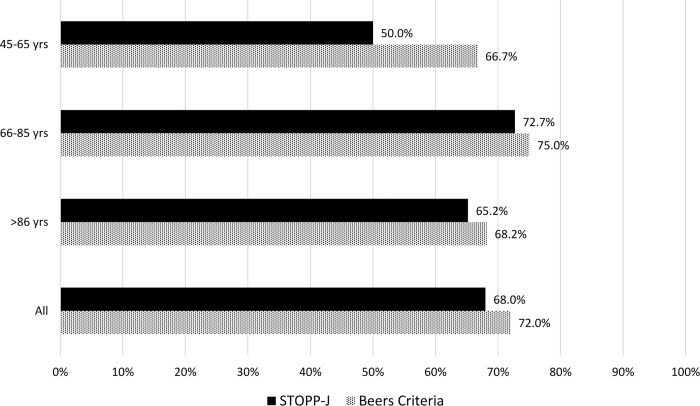
Proportion of PIM use by age.

**Fig 2:**
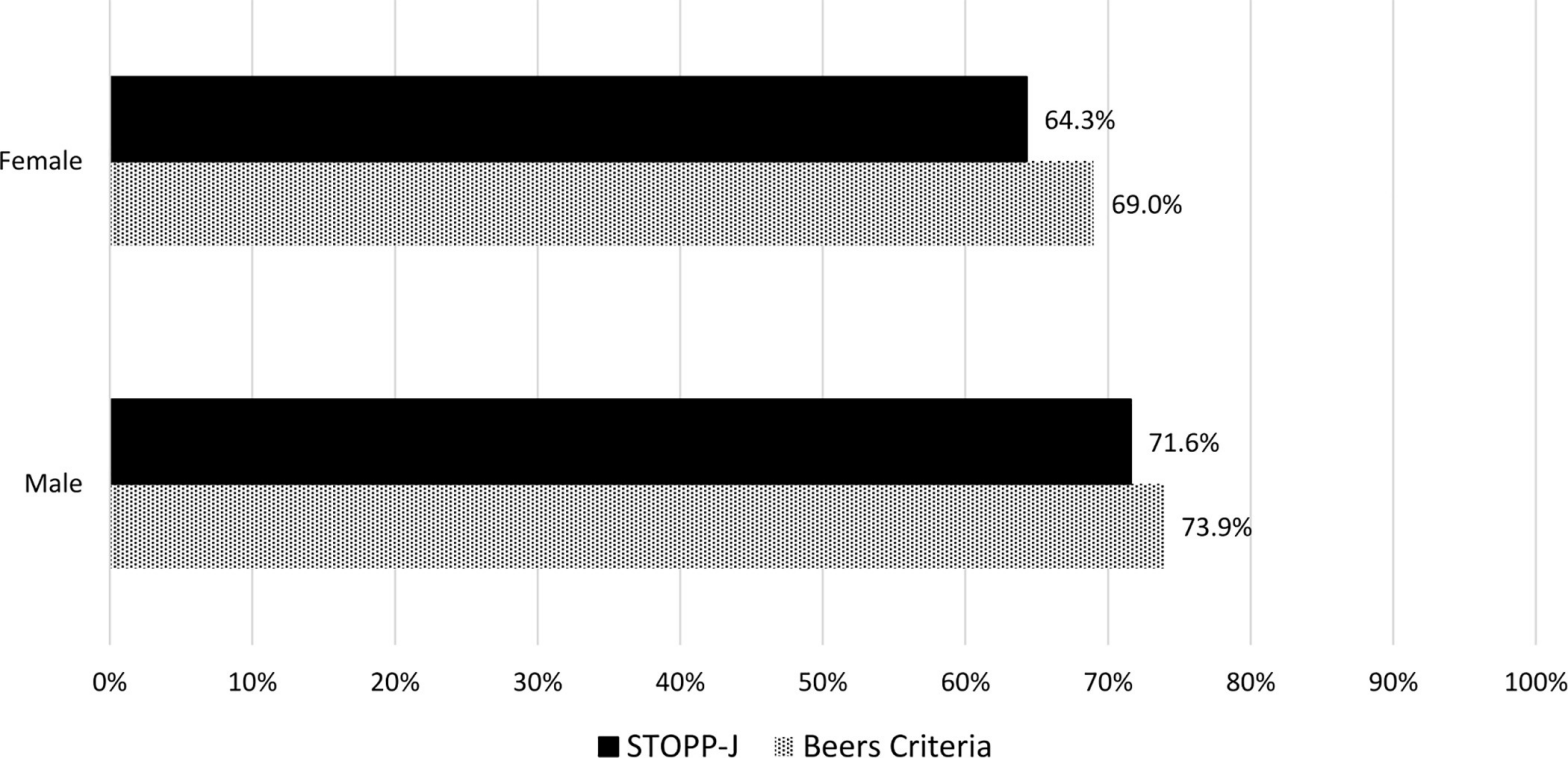
Proportion of PIM use by sex.

**Fig 3:**
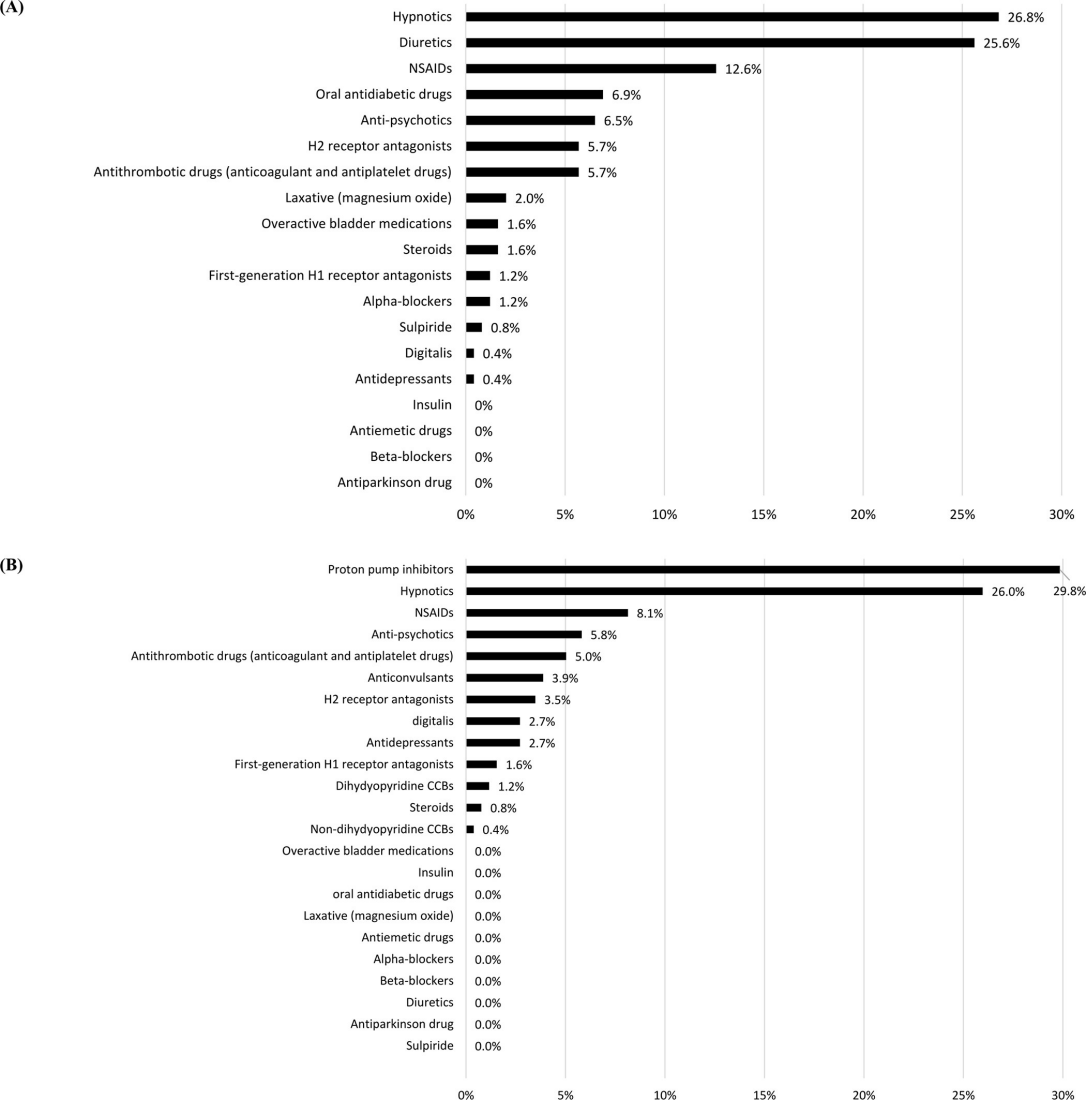
PIM categories according to the STOPP-J (A) and Beers Criteria (B).

PIMs were detected in 132 patients (67.3%) by the STOPP-J and in 141 patients (71.9%) by the Beers Criteria, and the mean numbers of PIMs were 1.3 ± 1.3 and 1.2 ± 1.1, respectively (Fig 1). The proportions of patients using PIMs categorized by age and sex did not differ significantly between the groups (Figs 1 and 2). The three most frequently prescribed STOPP-J PIMs were hypnotics (26.8%), diuretics (25.6%), and NSAIDs (12.6%) (Fig 3A), compared with PPIs (29.8%), hypnotics (26%), and NSAIDs (8.1%) according to the Beers Criteria (Fig 3B).

### Association between mortality and PIM use

**Fig 4:**
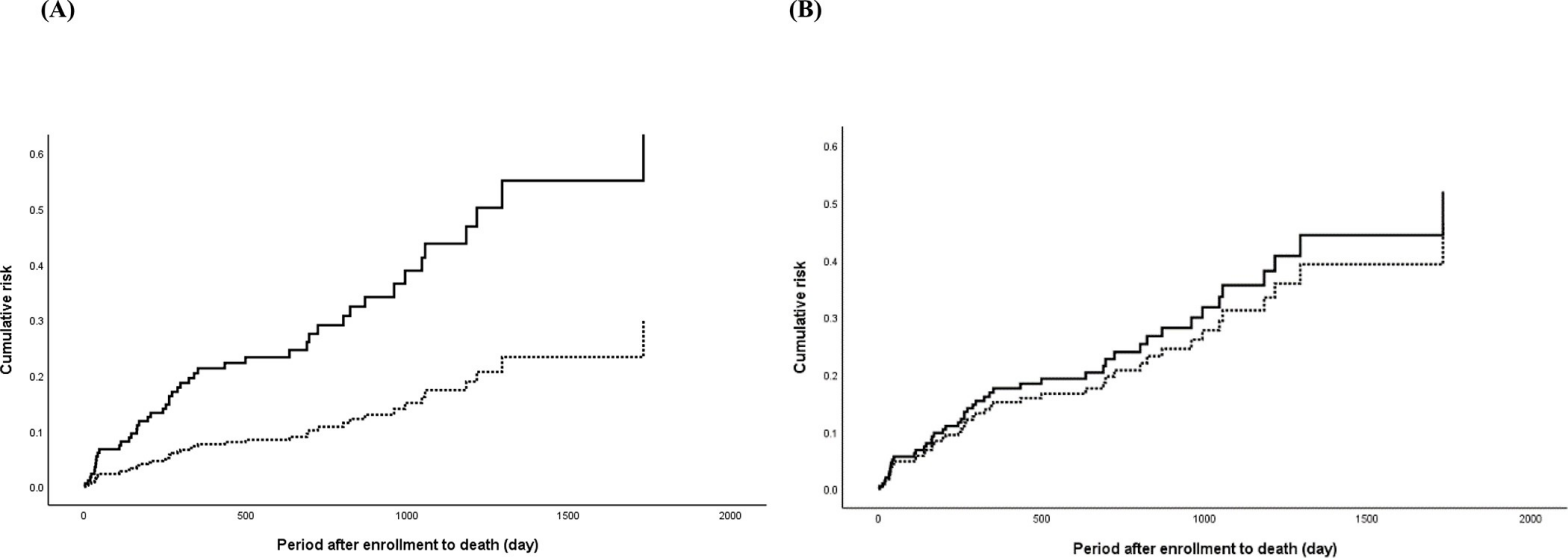
Cumulative risk of all-cause mortality for patients with PIM use (solid line) and without PIM use (dotted line) according to the STOPP-J (A) and Beers Criteria (B).

The Cox regression plot of all-cause mortality in patients with and without PIM use according to STOPP-J and the Beers Criteria is shown in Fig 4. In the multivariate Cox regression model, PIM use determined to STOPP-J had a significantly higher cumulative risk of all-cause mortality (HR 3.01, 95% CI 1.37–6.64) than those without PIM use after adjustment for covariates (Table 2). However, PIM use according to the Beers Criteria was not associated with all-cause mortality (Table 2). Multiple comorbidities, represented by CCI scores in our study, were associated with all-cause mortality (STOPP-J: HR 1.17, 95% CI 1.02–1.34; Beers Criteria: HR 1.17, 95% CI 1.02–1.35) (Table 2). In addition, a negative correlation was found between nutritional status measured by MNA-SF score and all-cause mortality (STOPP-J: HR 0.79, 95% CI 0.69–0.89; Beers Criteria: HR 0.81, 95% CI 0.72–0.92) (Table 2).

**Table 2:** Risk factors for all-cause mortality in a multivariate Cox regression model.

Variable	All-cause mortality							
	STOPP-J PIMs				Beers Criteria PIMs			
		95% CI				95% CI		
	HR	Lower limit	Upper limit	*p* value	HR	Lower limit	Upper limit	*p* value
Age (years)	1.03	0.99	1.06	0.13	1.03	1.00	1.07	0.09
Sex								
Female (versus male)	0.73	0.37	1.41	0.34	0.84	0.43	1.64	0.60
CCI score	**1.17**	**1.02**	**1.34**	**0.03**	**1.17**	**1.02**	**1.35**	**0.03**
Barthel Index score	1.00	0.99	1.01	0.51	1.00	0.99	1.01	0.65
MNA-SF score	**0.79**	**0.69**	**0.89**	**< 0.001**	**0.81**	**0.72**	**0.92**	**< 0.001**
Polypharmacy (≥5 medications)	0.72	0.37	1.39	0.33	1.05	0.53	2.09	0.88
PIM use								
No	0.00				0.00			
Yes	**3.01**	**1.37**	**6.64**	**0.01**	1.18	0.56	2.49	0.67

CCI, Charlson Comorbidity Index; Barthel Index of activities of daily living; MNA-SF, Mini Nutritional Assessment-Short Form.

### Association between hospitalization and PIM use

**Fig 5:**
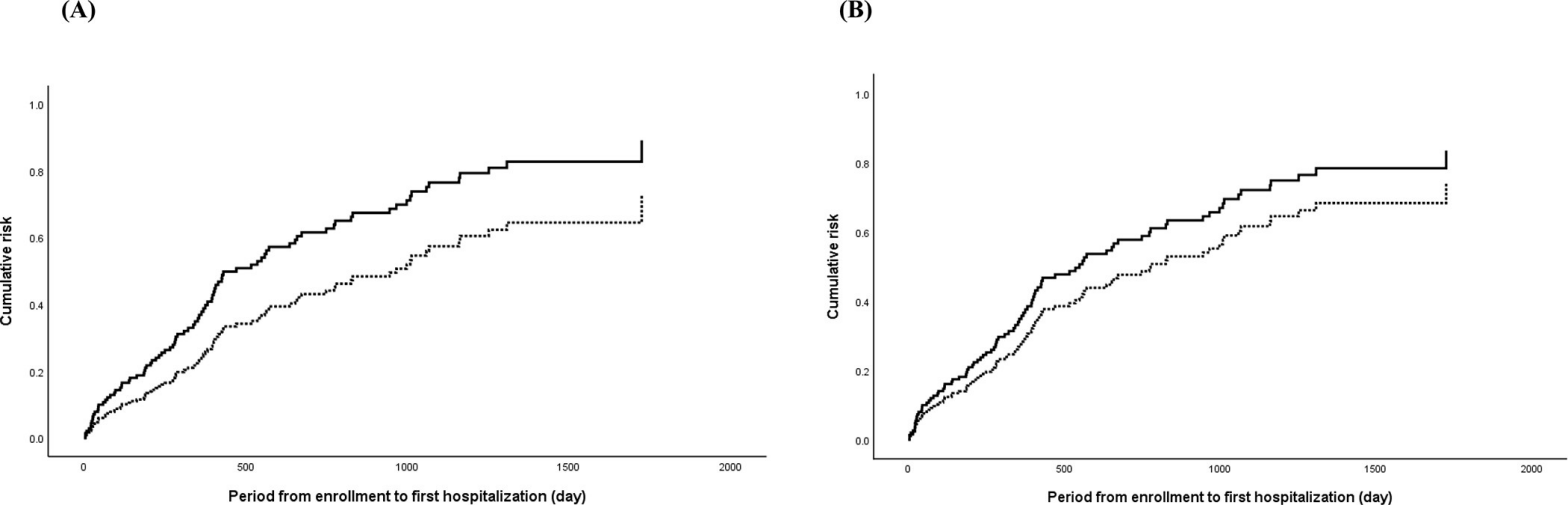
Cumulative risk of first hospitalization for patients with PIM use (solid line) and without PIM use (dotted line) according to the STOPP-J (A) and Beers Criteria (B).

The Cox regression plot of the risk of hospitalization in patients with and without PIM use according to STOPP-J and the Beers Criteria is shown in Fig 5. Similar to the results for all-cause mortality, only PIM use categorized by STOPP-J was associated with risk of hospitalization (HR 1.70, 95% CI 1.01–2.84) (Table 3); no association was found for PIM use categorized by the Beers Criteria. Neither multiple comorbidities nor malnutrition was correlated with risk of hospitalization.

**Table 3:** Risk factors for first hospitalization in a multivariate Cox regression model.

Variable	First hospitalization							
	STOPP-J PIMs				Beers Criteria PIMs			
		95% CI				95% CI		
	HR	Lower limit	Upper limit	*p* value	HR	Lower limit	Upper limit	*p* value
Age (years)	0.99	0.97	1.01	0.21	0.99	0.97	1.01	0.39
Sex								
Female (versus male)	1.11	0.69	1.78	0.66	1.13	0.71	1.81	0.61
CCI score	1.04	0.94	1.14	0.45	1.03	0.93	1.14	0.58
Barthel Index score	1.00	0.99	1.01	0.60	1.00	0.99	1.01	0.66
MNA-SF score	0.94	0.87	1.02	0.16	0.95	0.87	1.03	0.19
Polypharmacy (≥ 5 medications)	0.93	0.57	1.50	0.76	1.05	0.65	1.70	0.85
PIMs								
No								
Yes	**1.70**	**1.01**	**2.84**	**0.045**	1.33	0.77	2.31	0.31

CCI, Charlson Comorbidity Index; Barthel Index of activities of daily living; MNA-SF, Mini Nutritional Assessment-Short Form.

### Results of subgroup analysis

Because the major discriminant between the two criteria was PPIs, we performed a subgroup analysis using the modified Beers Criteria, which excludes PPIs from the original version. The results showed an association between the modified Beers Criteria and hospitalization (HR 1.91, 95% CI 1.17–3.09) but not mortality after controlling for age, sex, comorbidity, functional status, nutritional status, and polypharmacy (Table 4). On the other hand, because diuretics (loop diuretics and aldosterone antagonists), which are considered appropriate for patients with congestive heart failure (CHF), only appear in STOPP-J and not in the Beers Criteria, we further excluded patients with CHF for subgroup analysis. STOPP-J was still associated with mortality (HR 2.60, 95% CI 1.15–5.89) but not with hospitalization in patients without CHF (Table 4).

**Table 4:** Mortality and first hospitalization by different PIM criteria and patient subgroups.

PIM user	Mortality*				First hospitalization*			
		95% CI				95% CI		
	HR	Lower limit	Upper limit	*p* value	HR	Lower limit	Upper limit	*p* value
According to Beers Criteria	1.18	0.56	2.49	0.67	1.33	0.77	2.31	0.31
According to Modified Beers Criteria (excluding PPIs)	1.03	0.53	1.98	0.93	**1.91**	**1.17**	**3.09**	**0.01**
According to STOPP-J	**3.01**	**1.37**	**6.64**	**0.01**	**1.70**	**1.01**	**2.84**	**0.045**
According to STOPP-J (excluding patients with CHF)	**2.60**	**1.15**	**5.89**	**0.02**	1.70	0.98	2.94	0.06

CHF, congestive heart failure.

*Adjusted for age, sex, Charlson Comorbidity Index, Barthel Index, MNA-SF, and polypharmacy.

## Discussion

Because the consensus guidelines on PIMs in Japan (STOPP-J) were only released in 2016 [17], no other studies have compared them with the Beers Criteria to explore the relationship between PIMs and health outcomes in patients receiving home-based medical services. Although PIMs categorized by the Beers Criteria have been associated with admission, length of stay, and hospitalization [35], there is some discrepancy in drug classification and drug availability between the two sets of criteria. To the best of our knowledge, our study is the first to use STOPP-J to evaluate its ability to predict mortality and hospitalization rates in patients receiving home-based medical services.

PIMs, which are widespread in the elderly, were identified in more than 60% of patients in our study. This incidence in our home care setting was higher than that in community living centers [10] and comparable with that in aged care facilities [36, 37]. Consistent with the literature [10, 38], women were more likely and old-old individuals (age > 85 years) were less likely to be exposed to PIMs than men and young-old individuals (age 65–85 years). Therefore, young-old women, who are susceptible to potentially inappropriate prescribing, need periodic medication review as a high priority.

In this study, we compared an explicit international validated tool, the Beers Criteria, with STOPP-J, the recently updated Japanese version of STOPP. The incidence of PIMs according to STOPP-J is lower than that according to the Beers Criteria regardless of age and sex. Common PIMs listed in the two sets of criteria are hypnotics, NSAIDs, and antipsychotics, which are considered three of the most commonly prescribed PIMs [8, 39]. However, there are some discrepant categories in the initial ranks of common PIMs: PPIs, diuretics, and oral antidiabetic drugs (OADs).

PPIs are mainly used for gastric acid-related symptoms and diseases and possess superior efficacy and tolerability to histamine receptor antagonists and antacids. Nevertheless, PPIs increase the risks of fractures and *Clostridium difficile* infection [40, 41]. Therefore, both STOPP and the Beers Criteria include PPIs in their PIM lists [13, 15]. However, to prevent relapse and to prolong the remission period of gastroesophageal reflux disease [42], the Task Force of the Japanese Geriatric Society, which developed STOPP-J, chose to remove PPIs from the list. For this reason, PPIs, in the first rank of PIMs in the Beers Criteria, did not appear in the PIM list in STOPP-J that we used in the present study.

As for diuretics, loop diuretics and aldosterone antagonists are recommended to be limited to a lower dose by STOPP-J without consideration of indication or duration of use due to their frequent adverse effects, such as orthostatic hypotension, falls, and electrolyte imbalance, particularly hyperkalemia [17]. However, the Beers Criteria consider diuretics to be PIMs only when drug-drug interactions are encountered. For example, peripheral alpha-1 blockers are recommended not to be combined with loop diuretics due to an increased risk of urinary incontinence in women [15]. Additionally, angiotensin-converting enzyme inhibitors and potassium-sparing diuretics should not be combined except in the case of diagnosed systolic heart failure due to a higher risk of hyperkalemia. In short, due to aging-related homeostatic compromise, diuretics should be cautiously used as needed and maintained as low as possible in the elderly [43].

OADs are another class of drugs that may substantially contribute to harmful effects, including asymptomatic hypoglycemia and even coma [44]. Only sulfonylureas, which are notorious for higher risk of severe hypoglycemia, are included in the Beers Criteria. However, this category of drugs is nowadays seldom prescribed to elderly patients with diabetes [45]. On the other hand, aside from dipeptidyl peptidase-4 inhibitors, STOPP-J includes sulfonylureas, biguanides, thiazolidine derivatives, α-glucosidase inhibitors, and SGLT2 (sodium-glucose co-transporter-2) inhibitors due to the potential adverse effects of severe hypoglycemia, lactic acidosis, ileus, diarrhea, constipation, flatulence, dehydration, and urogenital infection [17]. The broader range of OADs accounted for the higher proportion of OADs in PIMs detected by STOPP-J than by the Beers Criteria in our sample.

To further clarify the effects of PIMs according to STOPP-J compared with those according to the Beers Criteria in patients receiving home-based medical services, we conducted an outcome-based evaluation using mortality and hospitalization. In Japan, one large-scale crossover longitudinal study using STOPP-J found that PIM use was correlated with a 1.5- to 4-fold increased risk of unexpected hospitalization [24]. In the present study, for disabled patients receiving home-based medical services, STOPP-J discriminated the risk of hospitalization and all-cause mortality among PIM and non-PIM users, unlike the Beers Criteria. These findings suggest that STOPP-J is a greater predictive modality for health outcomes than the Beers Criteria in community-dwelling disabled Japanese. Additionally, the relationship between the modified Beers Criteria and the risk of hospitalization implied that PPIs may not have deleterious effects on health outcomes in community-dwelling disabled Japanese. Hence, country-tailored criteria might play an important role in facilitating and promoting the clinical application of such criteria.

On the other hand, because diuretics can contribute to several complications, including fractures, falls, and hyperkalemia [46, 47], STOPP-J includes loop diuretics and aldosterone antagonists regardless of indication. However, in light of the recommended use of diuretics in patients with CHF in international guidelines [48, 49], there is a need to clarify the influence of this disease. In our non-CHF patients, PIM use according to STOPP-J was associated with high mortality. Although there is a discrepancy regarding decisions about diuretics between STOPP-J and the Beers Criteria, STOPP-J still provides mortality prediction.

This study has some limitations. First, drug compliance was not investigated thoroughly in the study. Patients needing home-based medical services are physically disabled and the inevitable polypharmacy would probably reduce adherence to medication. According to three large cohorts in Europe, adherence to medication in the elderly population is suboptimal for antihyperlipidemic, antiosteoporotic, and oral antidiabetic drugs [50]. Notably, these drugs are demonstrated to reduce cardiovascular events, hospitalization, and mortality [51–53]. In our study, however, leftover drugs were not counted and sorted at each visit. Future studies are warranted to monitor the status of unused and double-used drugs that might increase drug-drug interactions and result in fatal adverse effects. Secondly, Kampo, a combination of Japanese traditional herbal medicines extracted from plants and herbs, was not included in our PIM list. Kampo is used by the elderly as a supplementary treatment to maintain mental and physical wellbeing [54]. A retrospective cross-sectional study in Japan reported that *Glycyrrhizae radix*, the most commonly used component of Kampo, was potentially associated with chronic kidney disease and uncontrolled hypertension in elderly patients [55]. This unconfirmed relationship is worthy of further exploration and evaluation. Third, extrapolation of our results to the general population is not feasible. Studies focusing on community-dwelling outpatients could provide more comprehensive clinical implications.

A pharmacoeconomic model of PIM management is necessary. Previous studies reported that medication reviews may reduce PIM use and drug-related problems in aged care facilities, although the evidence is of low quality [56, 57]. One study suggested that an intervention model to manage PIMs in home-like care might reduce the costs of PIMs by 52% compared with standard group care in nursing homes [37]. Thus, a proactive recognition and response model should be developed in home-based and community-based care settings and investigated for clinical and cost-effectiveness. Further studies should also investigate whether medication reviews could reduce the prevalence and costs of PIMs.

On the other hand, although the use of explicit criteria such as STOPP-J and the Beers Criteria are potentially appropriate to guide physicians to identify and manage PIMs, care must be taken to ensure that any alternative prescription does not compromise treatment effectiveness in clinical practice. In addition, with regard to drug adherence, it is difficult to replace a fix-dose combination drug with other individual drugs. Hence, country-oriented, medication-specific criteria would be of considerable clinical utility.

## Conclusion

PIMs categorized by STOPP-J are associated with hospitalization and mortality in Japanese patients receiving home-based medical services. PPIs, which are commonly used for acid-related diseases, do not seem to have deleterious effects on health outcomes. Country-oriented, medication-specific criteria would be clinically useful. Future studies are required to determine the optimal model for minimizing the burden of PIMs.

## References

[1] Nothelle SK, Sharma R, Oakes AH, Jackson M, Segal JB. Determinants of Potentially Inappropriate Medication Use in Long-Term and Acute Care Settings: A Systematic Review. J Am Med Dir Assoc. 2017;18(9):806 e1– e17. Epub 2017/08/03. 10.1016/j.jamda.2017.06.005 28764876 PMC5581209

[2] Chang CM, Liu PY, Yang YH, Yang YC, Wu CF, Lu FH. Use of the Beers criteria to predict adverse drug reactions among first-visit elderly outpatients. Pharmacotherapy. 2005;25(6):831–8. Epub 2005/06/02. . 15927902 10.1592/phco.2005.25.6.831

[3] Hyttinen V, Jyrkka J, Valtonen H. A Systematic Review of the Impact of Potentially Inappropriate Medication on Health Care Utilization and Costs Among Older Adults. Med Care. 2016;54(10):950–64. Epub 2016/07/02. 10.1097/MLR.0000000000000587 . 27367864

[4] Wang KN, Bell JS, Chen EYH, Gilmartin-Thomas JFM, Ilomaki J. Medications and Prescribing Patterns as Factors Associated with Hospitalizations from Long-Term Care Facilities: A Systematic Review. Drugs Aging. 2018;35(5):423–57. Epub 2018/03/28. 10.1007/s40266-018-0537-3 . 29582403

[5] Kristensen RU, Norgaard A, Jensen-Dahm C, Gasse C, Wimberley T, Waldemar G. Polypharmacy and Potentially Inappropriate Medication in People with Dementia: A Nationwide Study. J Alzheimers Dis. 2018;63(1):383–94. Epub 2018/03/27. 10.3233/JAD-170905 . 29578483

[6] Jeon HL, Park J, Han E, Kim DS. Potentially inappropriate medication and hospitalization/emergency department visits among the elderly in Korea. Int J Qual Health Care. 2018;30(1):50–6. Epub 2018/02/14. 10.1093/intqhc/mzx171 . 29438504

[7] Redston MR, Hilmer SN, McLachlan AJ, Clough AJ, Gnjidic D. Prevalence of Potentially Inappropriate Medication Use in Older Inpatients with and without Cognitive Impairment: A Systematic Review. J Alzheimers Dis. 2018;61(4):1639–52. Epub 2017/12/28. 10.3233/JAD-170842 . 29278890

[8] Stevenson DG, Decker SL, Dwyer LL, Huskamp HA, Grabowski DC, Metzger ED, et al Antipsychotic and benzodiazepine use among nursing home residents: findings from the 2004 National Nursing Home Survey. Am J Geriatr Psychiatry. 2010;18(12):1078–92. Epub 2010/09/03. 10.1097/JGP.0b013e3181d6c0c6 20808119 PMC3009456

[9] Holmes HM, Sachs GA, Shega JW, Hougham GW, Cox Hayley D, Dale W. Integrating palliative medicine into the care of persons with advanced dementia: identifying appropriate medication use. J Am Geriatr Soc. 2008;56(7):1306–11. Epub 2008/05/17. 10.1111/j.1532-5415.2008.01741.x . 18482301

[10] Dosa D, Cai S, Gidmark S, Thomas K, Intrator O. Potentially inappropriate medication use in veterans residing in community living centers: have we gotten better? J Am Geriatr Soc. 2013;61(11):1994–9. Epub 2013/11/14. 10.1111/jgs.12516 . 24219201

[11] Ozalas SM, Huang V, Brunetti L, Reilly T. Comparison of Two Versions of the Beers Criteria and Adverse Outcomes in Older Hospitalized Patients. Consult Pharm. 2017;32(12):752–63. Epub 2018/02/23. 10.4140/TCP.n.2017.752 . 29467068

[12] Kose E, Hirai T, Seki T, Hayashi H. Role of potentially inappropriate medication use in rehabilitation outcomes for geriatric patients after strokes. Geriatr Gerontol Int. 2018;18(2):321–8. Epub 2017/11/07. 10.1111/ggi.13187 . 29105246

[13] O'Mahony D, O'Sullivan D, Byrne S, O'Connor MN, Ryan C, Gallagher P. STOPP/START criteria for potentially inappropriate prescribing in older people: version 2. Age Ageing. 2015;44(2):213–8. Epub 2014/10/18. 10.1093/ageing/afu145 25324330 PMC4339726

[14] Renom-Guiteras A, Meyer G, Thurmann PA. The EU(7)-PIM list: a list of potentially inappropriate medications for older people consented by experts from seven European countries. Eur J Clin Pharmacol. 2015;71(7):861–75. Epub 2015/05/15. 10.1007/s00228-015-1860-9 25967540 PMC4464049

[15] By the American Geriatrics Society Beers Criteria Update Expert P. American Geriatrics Society 2015 Updated Beers Criteria for Potentially Inappropriate Medication Use in Older Adults. J Am Geriatr Soc. 2015;63(11):2227–46. Epub 2015/10/09. 10.1111/jgs.13702 . 26446832

[16] Motter FR, Fritzen JS, Hilmer SN, Paniz EV, Paniz VMV. Potentially inappropriate medication in the elderly: a systematic review of validated explicit criteria. Eur J Clin Pharmacol. 2018;74(6):679–700. Epub 2018/03/29. 10.1007/s00228-018-2446-0 . 29589066

[17] Kojima T, Mizukami K, Tomita N, Arai H, Ohrui T, Eto M, et al Screening Tool for Older Persons' Appropriate Prescriptions for Japanese: Report of the Japan Geriatrics Society Working Group on "Guidelines for medical treatment and its safety in the elderly". Geriatr Gerontol Int. 2016;16(9):983–1001. Epub 2016/09/07. 10.1111/ggi.12890 . 27594406

[18] Guyatt GH, Oxman AD, Vist GE, Kunz R, Falck-Ytter Y, Alonso-Coello P, et al GRADE: an emerging consensus on rating quality of evidence and strength of recommendations. BMJ. 2008;336(7650):924–6. Epub 2008/04/26. 10.1136/bmj.39489.470347.AD 18436948 PMC2335261

[19] Hyttinen V, Jyrkka J, Saastamoinen LK, Vartiainen AK, Valtonen H. The association of potentially inappropriate medication use on health outcomes and hospital costs in community-dwelling older persons: a longitudinal 12-year study. The European journal of health economics: HEPAC: health economics in prevention and care. 2018 Epub 2018/07/07. 10.1007/s10198-018-0992-0 . 29978444

[20] Muhlack DC, Hoppe LK, Weberpals J, Brenner H, Schottker B. The Association of Potentially Inappropriate Medication at Older Age With Cardiovascular Events and Overall Mortality: A Systematic Review and Meta-Analysis of Cohort Studies. J Am Med Dir Assoc. 2017;18(3):211–20. Epub 2017/01/31. 10.1016/j.jamda.2016.11.025 . 28131719

[21] Ferrah N, Lovell JJ, Ibrahim JE. Systematic Review of the Prevalence of Medication Errors Resulting in Hospitalization and Death of Nursing Home Residents. J Am Geriatr Soc. 2017;65(2):433–42. Epub 2016/11/22. 10.1111/jgs.14683 . 27870068

[22] Juola AL, Pylkkanen S, Kautiainen H, Bell JS, Bjorkman MP, Finne-Soveri H, et al Burden of Potentially Harmful Medications and the Association With Quality of Life and Mortality Among Institutionalized Older People. J Am Med Dir Assoc. 2016;17(3):276.e9–14. Epub 2016/01/26. 10.1016/j.jamda.2015.12.011 . 26805751

[23] Cross AJ, George J, Woodward MC, Ames D, Brodaty H, Wolfe R, et al Potentially Inappropriate Medication, Anticholinergic Burden, and Mortality in People Attending Memory Clinics. J Alzheimers Dis. 2017;60(2):349–58. Epub 2017/09/05. 10.3233/JAD-170265 . 28869467

[24] Sato I, Yamamoto Y, Kato G, Kawakami K. Potentially Inappropriate Medication Prescribing and Risk of Unplanned Hospitalization among the Elderly: A Self-Matched, Case-Crossover Study. Drug Saf. 2018 Epub 2018/05/02. 10.1007/s40264-018-0676-9 . 29714005

[25] Caughey GE, Barratt JD, Shakib S, Kemp-Casey A, Roughead EE. Medication use and potentially high-risk prescribing in older patients hospitalized for diabetes: a missed opportunity to improve care? Diabet Med. 2017;34(3):432–9. Epub 2016/05/03. 10.1111/dme.13148 . 27135418

[26] Gutierrez-Valencia M, Izquierdo M, Malafarina V, Alonso-Renedo J, Gonzalez-Glaria B, Larrayoz-Sola B, et al Impact of hospitalization in an acute geriatric unit on polypharmacy and potentially inappropriate prescriptions: A retrospective study. Geriatr Gerontol Int. 2017;17(12):2354–60. Epub 2017/04/20. 10.1111/ggi.13073 . 28422415

[27] Sevilla-Sanchez D, Molist-Brunet N, Amblas-Novellas J, Espaulella-Panicot J, Codina-Jane C. Potentially inappropriate medication at hospital admission in patients with palliative care needs. Int J Clin Pharm. 2017;39(5):1018–30. Epub 2017/07/27. 10.1007/s11096-017-0518-3 . 28744675

[28] Umegaki H, Asai A, Kanda S, Maeda K, Shimojima T, Nomura H, et al Factors associated with unexpected admissions and mortality among low-functioning older patients receiving home medical care. Geriatr Gerontol Int. 2017;17(10):1623–7. Epub 2017/01/07. 10.1111/ggi.12943 . 28060439

[29] Tamiya N, Noguchi H, Nishi A, Reich MR, Ikegami N, Hashimoto H, et al Population ageing and wellbeing: lessons from Japan's long-term care insurance policy. Lancet. 2011;378(9797):1183–92. Epub 2011/09/03. 10.1016/S0140-6736(11)61176-8 . 21885099

[30] Charlson ME, Pompei P, Ales KL, MacKenzie CR. A new method of classifying prognostic comorbidity in longitudinal studies: development and validation. J Chronic Dis. 1987;40(5):373–83. Epub 1987/01/01. . 3558716 10.1016/0021-9681(87)90171-8

[31] Sato S, Demura S, Minami M, Kasuga K. Longitudinal assessment of ADL ability of partially dependent elderly people: examining the utility of the index and characteristics of longitudinal change in ADL ability. J Physiol Anthropol Appl Human Sci. 2002;21(4):179–87. Epub 2002/11/01. . 12407986 10.2114/jpa.21.179

[32] Rubenstein LZ, Harker JO, Salva A, Guigoz Y, Vellas B. Screening for undernutrition in geriatric practice: developing the short-form mini-nutritional assessment (MNA-SF). J Gerontol A Biol Sci Med Sci. 2001;56(6):M366–72. Epub 2001/05/31. . 11382797 10.1093/gerona/56.6.m366

[33] Kuzuya M, Kanda S, Koike T, Suzuki Y, Satake S, Iguchi A. Evaluation of Mini-Nutritional Assessment for Japanese frail elderly. Nutrition. 2005;21(4):498–503. Epub 2005/04/07. 10.1016/j.nut.2004.08.023 . 15811771

[34] Tokuda Y. Polypharmacy, Inappropriate Prescribing and Adverse Drug Events in Japan. Journal of General and Family Medicine. 2016;17(1):3–4.

[35] Grace AR, Briggs R, Kieran RE, Corcoran RM, Romero-Ortuno R, Coughlan TL, et al A comparison of beers and STOPP criteria in assessing potentially inappropriate medications in nursing home residents attending the emergency department. J Am Med Dir Assoc. 2014;15(11):830–4. Epub 2014/10/12. 10.1016/j.jamda.2014.08.008 . 25304180

[36] Vieira de Lima TJ, Garbin CA, Garbin AJ, Sumida DH, Saliba O. Potentially inappropriate medications used by the elderly: prevalence and risk factors in Brazilian care homes. BMC Geriatr. 2013;13:52 Epub 2013/05/31. 10.1186/1471-2318-13-52 23718678 PMC3679980

[37] Harrison SL, Kouladjian O'Donnell L, Milte R, Dyer SM, Gnanamanickam ES, Bradley C, et al Costs of potentially inappropriate medication use in residential aged care facilities. BMC Geriatr. 2018;18(1):9 Epub 2018/01/13. 10.1186/s12877-018-0704-8 29325531 PMC5765623

[38] Giovannini S, van der Roest HG, Carfi A, Finne-Soveri H, Garms-Homolova V, Declercq A, et al Polypharmacy in Home Care in Europe: Cross-Sectional Data from the IBenC Study. Drugs Aging. 2018;35(2):145–52. Epub 2018/02/08. 10.1007/s40266-018-0521-y . 29411310

[39] Nam YS, Han JS, Kim JY, Bae WK, Lee K. Prescription of potentially inappropriate medication in Korean older adults based on 2012 Beers Criteria: a cross-sectional population based study. BMC Geriatr. 2016;16:118 Epub 2016/06/04. 10.1186/s12877-016-0285-3 27255674 PMC4890525

[40] Schoenfeld AJ, Grady D. Adverse Effects Associated With Proton Pump Inhibitors. JAMA Intern Med. 2016;176(2):172–4. Epub 2016/01/12. 10.1001/jamainternmed.2015.7927 . 26751904

[41] Wilhelm SM, Rjater RG, Kale-Pradhan PB. Perils and pitfalls of long-term effects of proton pump inhibitors. Expert Rev Clin Pharmacol. 2013;6(4):443–51. Epub 2013/08/10. 10.1586/17512433.2013.811206 . 23927671

[42] Katz PO, Gerson LB, Vela MF. Guidelines for the diagnosis and management of gastroesophageal reflux disease. Am J Gastroenterol. 2013;108(3):308–28; quiz 29. Epub 2013/02/20. 10.1038/ajg.2012.444 . 23419381

[43] Khow K, Lau S, Li J, Yong T. Diuretic-Associated Electrolyte Disorders in the Elderly: Risk Factors, Impact, Management and Prevention. Current Drug Safety. 2014;9(1):2–15. 10.2174/1574886308666140109112730 24410347

[44] Quilliam BJ, Ozbay AB, Sill BE, Kogut SJ. The association between adherence to oral anti-diabetic drugs and hypoglycaemia in persons with Type 2 diabetes. Diabet Med. 2013;30(11):1305–13. Epub 2013/04/17. 10.1111/dme.12217 . 23586474

[45] Tirmizi S, Mazzola N. Review of safety considerations in the elderly using sulfonylureas. Consult Pharm. 2015;30(2):116–9. Epub 2015/02/20. 10.4140/TCP.n.2015.116 . 25695418

[46] Juurlink DN, Mamdani MM, Lee DS, Kopp A, Austin PC, Laupacis A, et al Rates of hyperkalemia after publication of the Randomized Aldactone Evaluation Study. N Engl J Med. 2004;351(6):543–51. Epub 2004/08/06. 10.1056/NEJMoa040135 . 15295047

[47] Lim LS, Fink HA, Blackwell T, Taylor BC, Ensrud KE. Loop diuretic use and rates of hip bone loss and risk of falls and fractures in older women. J Am Geriatr Soc. 2009;57(5):855–62. Epub 2009/04/17. 10.1111/j.1532-5415.2009.02195.x 19368583 PMC2721719

[48] Ponikowski P, Voors AA, Anker SD, Bueno H, Cleland JG, Coats AJ, et al 2016 ESC Guidelines for the diagnosis and treatment of acute and chronic heart failure: The Task Force for the diagnosis and treatment of acute and chronic heart failure of the European Society of Cardiology (ESC). Developed with the special contribution of the Heart Failure Association (HFA) of the ESC. European journal of heart failure. 2016;18(8):891–975. Epub 2016/05/22. 10.1002/ejhf.592 . 27207191

[49] Reboussin DM, Allen NB, Griswold ME, Guallar E, Hong Y, Lackland DT, et al Systematic Review for the 2017 ACC/AHA/AAPA/ABC/ACPM/AGS/APhA/ASH/ASPC/NMA/PCNA Guideline for the Prevention, Detection, Evaluation, and Management of High Blood Pressure in Adults: A Report of the American College of Cardiology/American Heart Association Task Force on Clinical Practice Guidelines. Circulation. 2018;138(17):e595–e616. Epub 2018/10/26. 10.1161/CIR.0000000000000601 . 30354656

[50] Menditto E, Cahir C, Aza-Pascual-Salcedo M, Bruzzese D, Poblador-Plou B, Malo S, et al Adherence to chronic medication in older populations: application of a common protocol among three European cohorts. Patient preference and adherence. 2018;12:1975–87. Epub 2018/10/17. 10.2147/PPA.S164819 30323567 PMC6179242

[51] Savarese G, Gotto AM Jr., Paolillo S, D'Amore C, Losco T, Musella F, et al Benefits of statins in elderly subjects without established cardiovascular disease: a meta-analysis. Journal of the American College of Cardiology. 2013;62(22):2090–9. Epub 2013/08/21. 10.1016/j.jacc.2013.07.069 . 23954343

[52] Fei Y, Tsoi MF, Kumana CR, Cheung TT, Cheung BMY. Network meta-analysis of cardiovascular outcomes in randomized controlled trials of new antidiabetic drugs. International journal of cardiology. 2018;254:291–6. Epub 2017/12/27. 10.1016/j.ijcard.2017.12.039 . 29277321

[53] Hawley S, Javaid MK, Prieto-Alhambra D, Lippett J, Sheard S, Arden NK, et al Clinical effectiveness of orthogeriatric and fracture liaison service models of care for hip fracture patients: population-based longitudinal study. Age Ageing. 2016;45(2):236–42. Epub 2016/01/24. 10.1093/ageing/afv204 ; PubMed Central PMCID: PMCPMC4776625. 26802076 PMC4776625

[54] Nakae H, Hiroshima Y, Hebiguchi M. Kampo Medicines for Frailty in Locomotor Disease. Front Nutr. 2018;5:31 Epub 2018/05/15. 10.3389/fnut.2018.00031 29755984 PMC5933258

[55] Komagamine J, Hagane K. Prevalence of the potentially inappropriate Kampo medications to be used with caution among elderly patients taking any prescribed Kampo medications at a single centre in Japan: a retrospective cross-sectional study. BMC Complement Altern Med. 2018;18(1):155 Epub 2018/05/13. 10.1186/s12906-018-2228-3 29751840 PMC5948909

[56] Forsetlund L, Eike MC, Gjerberg E, Vist GE. Effect of interventions to reduce potentially inappropriate use of drugs in nursing homes: a systematic review of randomised controlled trials. BMC Geriatr. 2011;11:16 Epub 2011/04/19. 10.1186/1471-2318-11-16 21496345 PMC3108292

[57] Gheewala PA, Peterson GM, Curtain CM, Nishtala PS, Hannan PJ, Castelino RL. Impact of the pharmacist medication review services on drug-related problems and potentially inappropriate prescribing of renally cleared medications in residents of aged care facilities. Drugs Aging. 2014;31(11):825–35. Epub 2014/09/05. 10.1007/s40266-014-0208-y . 25187228

